# BRAF Inhibitor Resistance Confers Increased Sensitivity to Mitotic Inhibitors

**DOI:** 10.3389/fonc.2022.766794

**Published:** 2022-04-04

**Authors:** Sean A. Misek, Bardees M. Foda, Thomas S. Dexheimer, Maisah Akram, Susan E. Conrad, Jens C. Schmidt, Richard R. Neubig, Kathleen A. Gallo

**Affiliations:** ^1^ Department of Physiology, Michigan State University, East Lansing, MI, United States; ^2^ Department of Pharmacology and Toxicology, Michigan State University, East Lansing, MI, United States; ^3^ Molecular Genetics and Enzymology Department, National Research Centre, Dokki, Egypt; ^4^ Department of Microbiology and Molecular Genetics, Michigan State University, East Lansing, MI, United States; ^5^ Department of Obstetrics, Gynecology and Reproductive Biology, Michigan State University, East Lansing, MI, United States; ^6^ Institute for Quantitative Health Science and Engineering, Michigan State University, East Lansing, MI, United States; ^7^ “Nicholas V. Perricone, M.D.”, Division of Dermatology, Department of Medicine, Michigan State University, East Lansing, MI, United States

**Keywords:** BRAF, resistance, mitosis, inhibitor, pharmacology, compound screen, melanoma, vemurafenib

## Abstract

Single agent and combination therapy with BRAF^V600E/K^ and MEK inhibitors have remarkable efficacy against melanoma tumors with activating BRAF mutations, but in most cases BRAF inhibitor (BRAFi) resistance eventually develops. One resistance mechanism is reactivation of the ERK pathway. However, only about half of BRAFi resistance is due to ERK reactivation. The purpose of this study is to uncover pharmacological vulnerabilities of BRAFi-resistant melanoma cells, with the goal of identifying new therapeutic options for patients whose tumors have developed resistance to BRAFi/MEKi therapy. We screened a well-annotated compound library against a panel of isogenic pairs of parental and BRAFi-resistant melanoma cell lines to identify classes of compounds that selectively target BRAFi-resistant cells over their BRAFi-sensitive counterparts. Two distinct patterns of increased sensitivity to classes of pharmacological inhibitors emerged. In two cell line pairs, BRAFi resistance conferred increased sensitivity to compounds that share the property of cell cycle arrest at M-phase, including inhibitors of aurora kinase (AURK), polo-like kinase (PLK), tubulin, and kinesin. Live cell microscopy, used to track mitosis in real time, revealed that parental but not BRAFi-resistant melanoma cells were able to exit from compound-induced mitotic arrest through mitotic slippage, thus escaping death. Consistent with the key role of Cyclin B1 levels in regulating mitosis at the spindle checkpoint in arrested cells, we found lower Cyclin B1 levels in parental compared with BRAFi-resistant melanoma cells, suggesting that inability to down-regulate Cyclin B1 expression levels may explain the increased vulnerability of resistant cells to mitotic inhibitors. Another BRAFi-resistant cell line showed increased sensitivity to Chk1/2 inhibitors, which was associated with an accumulation of DNA damage, resulting in mitotic failure. This study demonstrates that BRAFi-resistance, in at least a subset of melanoma cells, confers vulnerability to pharmacological disruption of mitosis and suggests a targeted synthetic lethal approach for overcoming resistance to BRAF/MEK-directed therapies.

## Introduction

Many mechanisms of BRAFi/MEKi resistance in BRAF-mutant melanoma are well understood (1–12), yet systematic approaches to identifying effective second-line therapies are still largely lacking. One appealing strategy to treat drug-resistant melanoma is to re-purpose drugs that have been FDA-approved for other indications since they can be quickly translated to the clinic. Large-scale efforts have sought to systematically profile compounds against annotated panels of cancer cell lines, initially with datasets like Genomics of Drug Sensitivity in Cancer (GDSC) (13) or Cancer Target Discovery and Development (CTD^2^) (14), and more recently with Profiling Relative Inhibition Simultaneously in Mixtures (PRISM) (15, 16). The ultimate goal of each of these initiatives is to correlate genomic features with drug responses and to map those associations back to patient tumors. Additional targeted screens have also been used to identify compounds with activity against drug-resistant cancer models (17–20).

The strategy we implemented was to screen a library of chemical compounds against pairs of isogenic parental and BRAFi-resistant melanoma cell lines. Chemical compound screens compare well with functional genomics-based CRISPR screens, but also present several distinct advantages. Most standard CRISPR screens are based upon perturbation of individual genes often leading to compensation by redundant isoforms, whereas compound screens typically contain inhibitors that target multiple members of the same protein family. Furthermore, CRISPR screens typically rely upon measurement of responses that require long-term deletion of a target gene. Thus, if a gene is essential for survival of all cells, it is impossible to assess the differential dependence of various cell populations on that gene. Finally, a drug repurposing approach immediately highlights promising candidates with activity against the target cells that could be translated to the clinic.

Mitotic inhibitors are often effective therapies for treating cancer as they induce mitotic arrest, followed by cell death. However, resistance to antimitotic therapies can occur when cancer cells undergo mitotic slippage, allowing them to them to survive in a polyploid state. Cyclin B1 levels are critical in regulating exit from mitotic arrest. Gradual degradation of Cyclin B1 during prolonged cell cycle arrest results in premature chromosome decondensation (21) and cells subsequently exit from the cell cycle into a 4n state. These cells are senescent, but under certain conditions such as loss of p53 they can re-enter into the cell cycle to give rise to polyploid cells (22).

In our screen, which was designed to reveal pharmacological vulnerabilities of BRAFi-resistant melanoma cells, we identified compounds that disrupt mitosis through multiple, distinct mechanisms. For instance, Aurora kinase (AURK) and Polo-like kinase (PLK) inhibitors as well as inhibitors of tubulin polymerization arrest cells in mitosis and prevent chromosome alignment during metaphase. These classes of compounds selectively induced prolonged cell cycle arrest and apoptosis in BRAFi-resistant cells. In contrast we found that the parental melanoma cells were markedly less sensitive to mitotic inhibitors. We further elucidated the mechanistic basis for this selectivity by demonstrating that after treatment with an AURK inhibitor, parental melanoma cells have a greater propensity to undergo mitotic slippage than their BRAFi resistant counterparts. Lower levels of Cyclin B1 were observed in parental cells compared with BRAFi resistant melanoma cells after treatment with AURK inhibitor. These findings are consistent with the model in which parental BRAF mutant melanoma cell lines retain the ability to degrade Cyclin B1 and thus evade mitotic inhibitor-induced death by undergoing mitotic slippage, whereas their BRAFi resistant counterparts are unable to downregulate Cyclin B1 and are subsequently die. Not all BRAFi resistant melanoma lines share this enhanced selectivity for these mitotic inhibitors. One of the screened BRAFi-resistant melanoma cell lines is more sensitive to pharmacological inhibition of Chk1/2 than its isogenic parental cell line. Our data suggest that this is due to increased accumulation of DNA damage which results in mitotic failure, and ultimately cell death.

In summary our work has identified new potential approaches to treating BRAFi resistant melanomas. Furthermore, we have probed two distinct mechanisms through which BRAFi-resistance in melanoma cells confers new vulnerability to pharmacological disruption of mitosis. From these studies emerges the exciting possibility that mitotic inhibitors may serve as potential new treatment strategies for BRAFi-resistant melanoma tumors. In addition, exploiting these vulnerabilities may be valuable in preventing the development of BRAFi resistance outright.

## Materials and Methods

### Cell Lines, Reagents, and Antibodies

Parental (denoted by a P suffix in the cell line name) and matched isogenic BRAFi-resistant cells (denoted by an R suffix in the cell line name) were either a gift from Dr. Roger Lo (UCLA) (M229P/R, M238P/R, or M249P/R) (7) or generated in our laboratory (UACC62P/R), as previously described (23).

BI-2536 (#17385), Volasertib (#18193), GSK461364 (#18099), Danusertib (#18387), AMG900 (#19176), MLN8237 (#13602), Docetaxel (#11637), Ispinesib (#18014), Mebendazole (#18872), AZD7762 (#11491), LY2603618 (#20351), SCH900776 (#18131), and Vemurafenib (#10618) were purchased from Cayman Chemical (Ann Arbor, USA). All compounds were diluted in DMSO to a stock concentration of 10 mM and aliquots were stored at -20°C. Antibodies against γH2AX (#9718), PARP (#9542), Cyclin A2 (#67955), and Cyclin B1 (#12231) were purchased from Cell Signaling Technology (Danvers, USA). Alexa Fluor488 goat anti-rabbit (#A11034) was purchased from Invitrogen (Carlsbad, USA). Recombinant human TNFα protein (#210-TA-005) was purchased from R&D Systems (Minneapolis, USA).

Cells were cultured in DMEM (ThermoFisher, Waltham, USA #11995-065) supplemented with 10% fetal bovine serum (FBS) (ThermoFisher, #10437-028) and 1% Antibiotic-Antimycotic (ThermoFisher, #15240062) and were passaged at approximately 75% confluence. The BRAFi-resistant cell line variants were maintained in culture medium supplemented with 2 µM vemurafenib. Vemurafenib was removed from the culture medium when cells were seeded for experiments, except where otherwise indicated. Cells were routinely tested for mycoplasma contamination by DAPI staining. Short Tandem Repeat profiling of all cell lines was performed at the MSU genomics core. In all cases, isogenic pairs of cell lines had identical STR profiles.

### Viability Experiments

Cells were seeded into white 384-well tissue culture plates (PerkinElmer, Waltham, USA, #6007689) at a density of 1000 cells/well in 20 µL of growth medium. The next day, compounds were pre-diluted in growth medium and then added to the 384-well plates so that the final volume of each well was 40 µL. The outer wells of the plate received no cells, and a PBS or growth medium barrier was added to those wells to limit evaporation from wells containing cells. Cells were cultured under these conditions for 72 h. To assess viability, 8 µL of CellTiter-Glo (Promega, Madison, USA, #G7573) was added to each well. Plates were incubated on an orbital shaker for 5 min at room temperature, then briefly centrifuged (4000 rpm, 60 s) before being read on a Bio-Tek Synergy Neo plate reader with the #11 and #41 Ex/Em filter cubes. Viability signal was plotted versus the log of the vemurafenib concentration for each treatment condition.

### Compound Library Screen

Cells were seeded into white 384-well plates at a density of 1,000 cells/well. The next day the NCATS MIPe chemical library of 1910 compounds (24) was added to the plates with a 50 nl pin tool at a final concentration of 200 nM. After 72 h, 8 µL of CellTiter-Glo was added to each well. The plates were incubated on an orbital shaker for 5 min, briefly spun down, and cell viability was measured as described above. In some cases, noise in the assay produced viability measurements that were greater than 100%. In these situations, the viability measurement was set to 100%.

### Cell Cycle Analysis

Cells were rinsed once in PBS, incubated with trypsin, washed once in PBS and immediately fixed in 70% ethanol for 20 min at room temperature. The cells were washed once and were re-suspended in PBS supplemented with 20 µg/mL propidium iodide (#P1304MP, ThermoFisher) and 200 µg/mL RNaseA. The cells were briefly mixed and were incubated on ice for 20 min. Following incubation, the cells were filtered through a 70 µM filter and were run on an Accuri C6 flow cytometer (BD Biosciences, Franklin Lakes, USA). Data were analyzed with the FCS Express flow cytometry analysis software package.

### Generation of Recombinant Constructs

Scarlet-H2A was amplified using PCR (donor plasmid: Addgene #85051, from Dorus Gadella) and subcloned into pDONR221 using the Gateway BP Clonase II enzyme mix (#11789020) from ThermoFisher. It was subsequently subcloned into the pLX301 lentiviral expression vector (from David Root, Addgene plasmid #25895, RRID : Addgene_25895) using the Gateway LR Clonase II enzyme mix (#11791020) from ThermoFisher. TUBA1B was amplified using PCR (donor plasmid: Addgene #57159, RRID : Addgene_25897, from Michael Davidson) and an EGFP-TUBA1B fusion protein was generated with two-stage overhang extension PCR using the TUBA1B and EGFP cDNA fragments. The EGFP-TUBA1B fusion protein was subcloned into pDONR221 and was subsequently cloned into pLX303 (from David Root, Addgene #25897, RRID : Addgene_48993). CyclinB1-GFP was amplified using PCR (donor plasmid: Addgene #26061, from Jonathon Pines) and was subcloned into pDONR221 (RRID : Addgene_127644) and subsequently subcloned into pLX303. All PCR primers are listed in **Supplementary Table S1**. Successful cloning was confirmed by Sanger sequencing.

### Virus Generation and Infection

HEK-293T cells were seeded onto 10-cm plates at a density of 4x10^6^ cells/plate and the cells were allowed to attach overnight. The next day the cells were transfected with a plasmid cocktail containing 5000 ng of the pLentiCRISPRv2 plasmid, 5000 ng of psPAX2 (Addgene plasmid #12260, RRID : Addgene_12260), 500 ng of pMD2.G (Addgene plasmid #12259, RRID : Addgene_12259), and 20 µL of Lipofectamine 2000 (ThermoFisher, #11668019) in 400 µL of OptiMEM (ThermoFisher, #31985070). The next morning the medium was changed to 10 mL of fresh complete culture medium, and the following day each plate was supplemented with an additional 5 mL of culture medium. After 24 h, the culture medium was harvested and filtered through a 0.45-µm syringe filter. Virus was stored at 4°C and used within 2 weeks.

Melanoma cells were seeded onto 10-cm plates at a density of 5x10^5^ cells/plate in 10 mL of complete culture medium. Prior to adherence of cells, 3 mL of viral supernatant was added to each plate. The cells were incubated with virus for 24 h, then the medium was changed to 10 mL of fresh medium. After at least 7 days the cells were used in live cell imaging experiments without undergoing antibiotic selection.

### Assay for Reactive Oxygen Species

Cells were seeded at a density of 10,000 cells/well in a 96-well plate and allowed to attach overnight. The next day reactive oxygen species (ROS) levels were measured. The ROS assay (#MAK145, Sigma-Aldrich, St. Louis, USA) was performed as described in the manufacturer’s protocol for adherent cells. Cells were also treated with 1 mM H_2_O_2_ for 15 min as a positive control.

### Immunofluorescence Staining

Cells were seeded into 8-well chamber slides and were treated as indicated in the figure legends. Cells were fixed with 3.7% formaldehyde for 15 min then blocked in 2% BSA PBS-Triton X-100 (0.1%) for 1 h at room temperature. Cells were incubated overnight at 4°C in phospho-γH2AX antibody at a dilution of 1:1,000 in blocking buffer. Cells were washed thrice in PBS and then were incubated in the appropriate secondary antibody at a 1:1,000 dilution for 1 h at room temperature. Cells were washed 3 times in PBS and slides were then mounted in ProLong Gold Antifade + DAPI (ThermoFisher, #P36935). Slides were cured overnight at room temperature, and then transferred to 4°C. Slides were imaged on a Nikon TE2000-U fluorescence microscope at 20x magnification. All images were automatically quantified using an ImageJ (RRID : SCR_003070) pipeline. Briefly, nuclear masks were created from the DAPI channel and the phospho-γH2AX staining intensity was measured within each mask. Data is reported as relative phospho-γH2AX fluorescence intensity. At least 500 cells were quantified per treatment condition.

### Live Cell Imaging

To quantify the rate and outcome of mitosis in melanoma cells, UACC62P/R and M229P/R cells engineered to express Scarlet-H2A and EGFP-TUBA1B were utilized. Cells were seeded at a density of 5,000 per well in a glass-bottom 96-well plate. The next day the cells were treated as described in the figure legends and were imaged at 3-min intervals on a BioTek Cytation 3. Over 40 cells per treatment condition were analyzed for mitotic rate and outcome. The T_0_ for mitotic entry was defined as nuclear envelope breakdown and the final time was defined as either completion of mitosis (chromosome segregation and complete de-condensation), mitotic slippage (complete de-condensation of chromosomes), or prolonged arrest at the end of imaging.

To generate high resolution images, cells were seeded at a density of 10,000 per well in 8-well glass-bottom chamber slides. The next day the growth medium was changed to CO_2_-independent growth medium (Gibco, #18045088) and the cells were treated as described in the figure legends. Cells were imaged with a 20x air objective on a DeltaVision microscope equipped with an sCMOS camera, environmental chamber, and ultimate focus drift correction system. Five z-sections were imaged in 2 µm steps at 3-min time intervals. Equivalent exposure conditions were used for all images.

The described DeltaVision setup and imaging parameters were used to generate quantitative Cyclin B1 protein expression data. At least 10 cells were analyzed per treatment condition. Cyclin B1 expression was quantified at each time interval in with FIJI v1.52p (RRID : SCR_002285). Cyclin B1 expression was normalized to the expression value at the first analyzed timepoint.

## Results

### BRAFi-Resistant Melanoma Cells Are Sensitive to Inhibitors that Disrupt Mitosis

**Figure 1:**
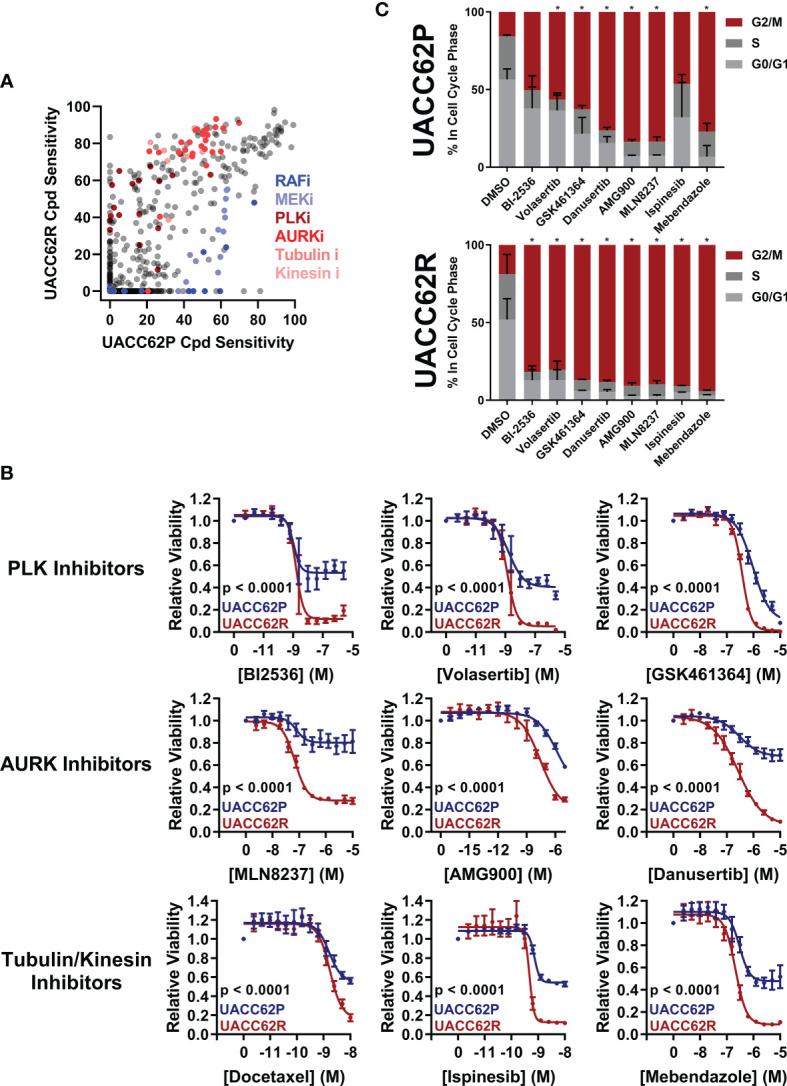
Vemurafenib-resistant UACC62R cells are selectively vulnerable to pharmacological disruption of mitosis. **(A)** The NCATS MIPe chemical library was screened against parental (P) and Vemurafenib-resistant UACC62R cells at 200 nM as described in the *Materials and Methods* section. Compound sensitivity data are plotted as % reduction in viability of UACC62P cells vs UACC62R cells for each compound in the screen. The larger the sensitivity value, the greater was the reduction in cell viability. The screen was performed with n = 1 replicates for each cell line. Inhibitors with selected targets are indicated in shades of *blue* for those that showed greater efficacy in UACC62P cells, and in shades of red for those that showed greater efficacy in UACC62R cells. **(B)** Fresh powder for selected compounds identified in the initial screen was obtained and the effect of these compounds on cell viability was analyzed at the indicated concentrations. *Blue* lines represent data for the UACC62P cells, and *red* lines indicate data for UACC62R cells. Data are represented as mean ± SE of the technical replicate averages (n = 3) for each of the biological replicates (n = 3). IC_50_ and E_max_ values are listed in **Supplementary Table S3**. **(C)** Cell cycle analyses of vehicle and drug-treated UACC62P/R cells were performed as described in the *Materials and Methods* section. All compounds were used at concentrations of 1 µM except for Ispinesib which was analyzed at 1 nM. Statistical analyses were performed on the proportion of cells in G2/M and in S phases for the drug-treated samples vs the DMSO control using one-way ANOVA analysis, * indicates p < 0.01. Data are represented as mean ± SE for n = 3 biological replicates.

In this study, we sought to identify compounds that selectively target BRAFi-resistant melanoma cells as potential therapeutic strategies and as a window to understanding mechanisms through which resistance arises. In our initial screen we profiled the NCATS Mechanism Interrogation Plate (MIPe) library of 1910 compounds (24) against a pair of matched isogenic parental and BRAFi-resistant melanoma cells, UACC62P which harbors the BRAF^V600E^ oncogene and its resistant counterpart UACC62R which was developed by *in vitro* selection with vemurafenib and is resistant to multiple BRAF inhibitors, including dabrafenib and encorafenib (23). The NCATS MIPe library contains a mechanistically and structurally diverse set of compounds, the majority of which are either FDA-approved or investigational new drugs and are directed at over 900 unique protein targets. The library is also redundant, containing multiple inhibitors against any one individual protein. This approach allows us to not only identify efficacious compounds, but also to gain new mechanistic insights into the molecular mechanisms of BRAFi resistance. **Figure 1A** shows a graphical representation of sensitivity of each compound against the UACC62P (x-axis) and UACC62R (y-axis) cell lines. As expected, unlike the parental counterpart, the UACC62R cells were insensitive to RAF and MEK inhibitors in this library, demonstrating that our screen can identify compounds that differ in their selectivity towards the parental BRAFi-sensitive and BRAFi-resistant melanoma cells. Among the top 25 compounds that selectively reduce viability of BRAFi-resistant UACC62R cells, we noticed a preponderance of mitotic inhibitors. As shown in **Figure 1A**, compounds that target PLK, AURK, tubulin, and kinesin selectively reduced viability of the UACC62R cells. To generalize these results, we evaluated the drug targets that are enriched across all 4 BRAFi resistant melanoma cell lines. We calculated the differential sensitivity for all 4 cell line pairs (R vs P) and identified any compound that showed a 20% greater cell viability reduction in at least one of the resistant cell lines. Raw viability data from the compound screen can be found in **Supplementary Table S2**. Of these, 179 compounds showed increased sensitivity by resistant cells, so we extracted the frequency of different drug targets represented by those compounds (**Table 1**). This confirmed the targets identified for the UACC62R cells but also identified topoisomerase, CDK, and Chk inhibitors as being enriched. Since the screen was performed at a single concentration of each compound, fresh powder was used to validate in concentration response studies of selected the mitotic inhibitor screen hits, which included 3 PLK inhibitors (BI2536, Volasertib, and GSK461364), 3 AURK inhibitors (Danusertib, AMG900, and MLN8237), 2 tubulin inhibitors (Docetaxel and Mebendazole), and the kinesin inhibitor Ispinesib. All these top hits were validated (**Figure 1B**). Interestingly, the differential compound sensitivity was found to be due to a change in the maximum percent inhibition (E_max)_, rather than due to a difference in the IC_50_ (**Figure 1B**). Our results indicate that mitotic blockade selectively reduces viability of BRAFi resistant UACC62 melanoma cells. No obvious synergy was observed between vemurafenib and any of the identified compounds (**Figure S1**) suggesting that alterations unique to the BRAFi resistant cells render them more vulnerable to disruption of mitosis.

**Table 1:** Frequency of target classes.

Rank	Target/Drug class	Freq	Short Target	Freq
1	Tubulin polymerization inhibitor	12	tubulin	22
2	Tubulin depolymerization inhibitor	9	aurora	15
3	Aurora-A/B/C Kinase Inhibitor	5	dna (topoisomerase)	15
4	DNA Topoisomerase II Inhibitors	5	cdk	6
5	Polo-like Kinase-1 (Plk-1) Inhibitor	5	chk	5
6	Aurora-A/B Inhibitor	4	polo	5
7	Chk1 Inhibitor	4	histone	4
8	DNA Topoisomerase I Inhibitors	4	jak	4
9	DNA Polymerase Inhibitors	3	antimitotic	3
10	Kinesin-Like Spindle Protein Inhibitor	3	kinesin	3

The cell growth inhibition data for the 1910 compounds on the NCATS MIPe plate for the 8 cell lines was evaluated for differential toxicity on the resistant vs. parental lines. The difference in sensitivity (DeltaSens = % inh R - % inh P) was calculated for the 4 pairs and the maximum value of the DeltaSens was determined. 179 compounds (~10% of the collection) had at least one cell line with a 20% increased sensitivity and were evaluated for target class frequency. Given multiple terms for the same target in the Target/Drug class list, another analysis calculated frequency on just the first word of drug class (Short Target). Data files are provided in the **Supplementary Table S2**.

We next focused on the two BRAF^V600E^ vemurafenib-sensitive/vemurafenib-resistant melanoma cell line pairs, M238P/R and M229P/R (7) which share a similar gene expression profile with the UACC62P/R cells. In particular, the resistant cells, compared with their vemurafenib-sensitive parental counterparts, lack expression of differentiation-associated melanocyte lineage genes (23). M238R cells showed a compound sensitivity pattern similar to UACC62R, with top hits including AURK inhibitors (**Figure S2**). Supporting the enrichment analysis in **Table 1**, the M229R cells showed increased sensitivity to Chk1/2 inhibitors over its parental BRAFi sensitive melanoma line (**Figure S2**). Interestingly, the identified AURK inhibitors that selectively target UACC62R over UACC62P cells were different from those that target M238R over M238P cells (**Supplementary Table S2**). Differences in expression or activities of drug efflux pumps or drug metabolizing enzymes expressed in the various BRAFi resistant melanoma lines could provide an explanation for these findings. We also evaluated the M249P and M249R melanoma pair. The M249R cells are unlike the other three resistant cell lines, which show increased activity of RhoA/C mechanisms and share a similar gene expression profile consistent with de-differentiation from the melanocytic pattern (23). The M249R cells acquired an activating NRAS^Q61^ mutation and have resistance due to reactivation of the ERK/MAPK pathway (7). In our screen, there was no enrichment of mitotic inhibitors with selectivity towards M249R cells, suggesting that mitotic inhibitors may be selective for BRAFi cells that do not show ERK/MAPK reactivation (**Figure S2** and **Supplementary Table S2**).

PLK, AURK, tubulin, and kinesin are all critical for the execution of mitosis, so we reasoned that altered regulation of mitosis might provide the mechanistic basis for the differences in compound selectivity between the UACC62P and UACC62R cells. Therefore, we performed cell cycle analyses to determine the impact of the mitotic inhibitors identified in our screen on cell cycle distribution of UACC62P and UACC62R cells. These data show that treatment with mitotic inhibitors results in a greater fraction of UACC62R cells with 4n DNA content (G2/M) compared with UACC62P, indicating that the UACC62R cells undergo more efficient mitotic arrest in response to drug treatment than their parental counterpart (**Figure 1C**).

### BRAFi-Sensitive, but Not BRAFi-Resistant, Melanoma Cells Undergo Mitotic Slippage

Our data demonstrate that a subset of BRAFi-resistant cell lines is more sensitive than the parental counterparts to inhibitors which disrupt mitosis. However, the mechanism behind this increased sensitivity was initially unclear. We first hypothesized that there might be increased levels of DNA damage in BRAFi-resistant cells which could enhance their sensitivity to pharmacological disruption of mitosis. However, neither ROS, which could in principle induce DNA damage, nor γH2AX staining, a marker of DNA damage, was elevated in UACC62R cells over levels in UACC62P cells (**Figures S3, S4**). Our previous studies showed that, compared with their BRAFi-sensitive counterparts, UACC62R melanoma cells express genes associated with de-differentiation (23). To investigate whether the lower sensitivity of UACC62P cells to mitotic inhibitors might be attributed to their more differentiated state compared with UACC62R cells, we treated both UACC62P and M229P cells with tumor necrosis factor α (TNFα) which has been shown to induce de-differentiation of melanoma cells (25, 26), and assessed the impact on sensitivity to a panel of mitotic inhibitors (**Figure S5**). The lack of effect of TNFα on sensitivity to mitotic inhibitors suggests that the de-differentiation attributes of the BRAFi-resistant cells do not fully explain their vulnerability to mitotic inhibitors (**Figure S5**).

**Figure 2:**
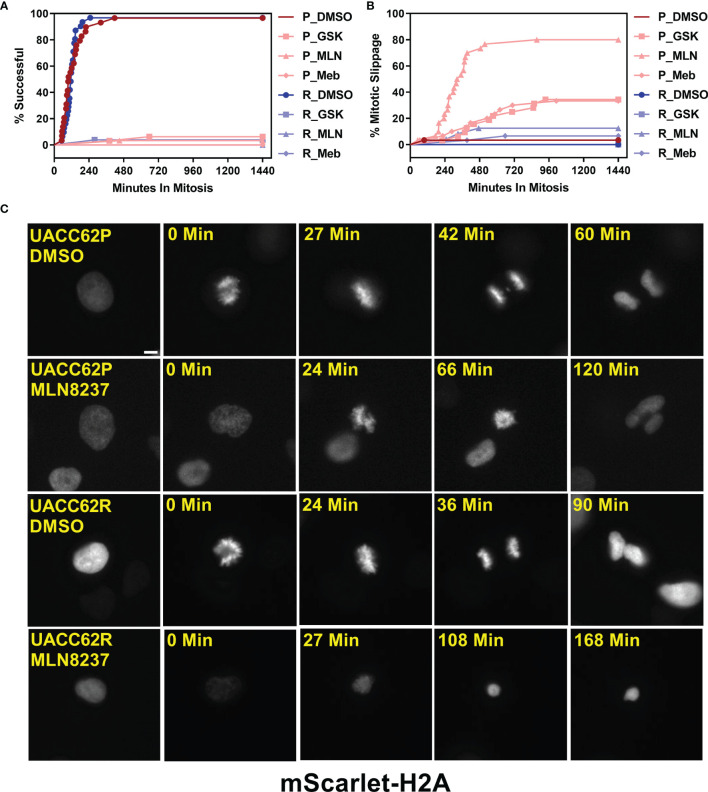
Compound-treated UACC62P, but not UACC62R, cells undergo mitotic slippage. UACC62P/R cells were engineered to stably express GFP-TUBA1B and mScarlet-H2A. The cells were seeded into glass-bottom 96-well plates and the next day the cells were treated with 1 µM GSK461364, MLN8237, or Mebendazole. Mitotic timing and outcomes were analyzed as described in *Materials and Methods*. The fraction of cells which **(A)** successfully completed mitosis or **(B)** underwent mitotic slippage is plotted as a function of time. At least 40 cells were analyzed per treatment condition. **(C)** Representative images of DMSO or MLN8237-treated UACC62P/R cells. The relative timepoints represent the time between nuclear envelope breakdown and the terminal mitotic event (either successful mitosis, mitotic arrest, or aberrant mitosis). Images were captured using the DeltaVision microscopy setup as described in the *Materials and Methods* section. Scale bar = 10 µM.

To evaluate how mitosis is affected in BRAFi-resistant and isogenic parental cells treated with or without mitotic inhibitors, we used live cell imaging. Fusion proteins of enhanced green fluorescent protein with the α-tubulin β chain (EGFP-TUBA1B) and of a red fluorescent protein with histone H2A (mScarlet-H2A) were used to label the mitotic spindle and chromosomes, respectively. Our initial hypothesis was that mitotic integrity in UACC62R cells might already be impaired under basal conditions, rendering them more vulnerable to pharmacological disruption of mitosis than the non-resistant parental cells. However, DMSO-treated UACC62P and UACC62R cells had similar mitotic timing duration and success rates, which was measured by tracking mScarlet-H2A localization as described in *Materials and Methods* (**Figure 2A**). In contrast to the differential effects of treatment of R and P cells with GSK461364 (PLKi), MLN8237 (AURKi), or Mebendazole (tubulin polymerization inhibitor) on cell viability (**Figure 1B**), compound treatment prevented the majority of both UACC62P and UACC62R cells from successfully completing mitosis (**Figure 2A**). Interestingly, a significant fraction of the compound-treated UACC62P cells initially arrested in mitosis, but after several hours underwent mitotic slippage (pink lines in **Figure 2B**, images in **Figure 2C**). In contrast, the resistant UACC62R cells rarely showed mitotic slippage (pale blue lines in **Figure 2B**, images in **Figure 2C**). The compound-treated cells appeared to arrest prior to metaphase; while there was chromosome condensation, the chromosomes did not successfully align at the metaphase plate in any of the parental or resistant cells (**Figure 2C**). The proportion of cells that underwent mitotic slippage in response to mitotic inhibitor drug treatment inversely correlated with the drug-induced decrease in viability (**Figure 1B**), suggesting that the inability of the resistant cells to undergo mitotic slippage is the basis of the selectivity of mitotic disrupters for BRAFi resistant UACC62R cells.

### Differential Cyclin B1 Accumulation in UACC62P/R Cells

**Figure 3:**
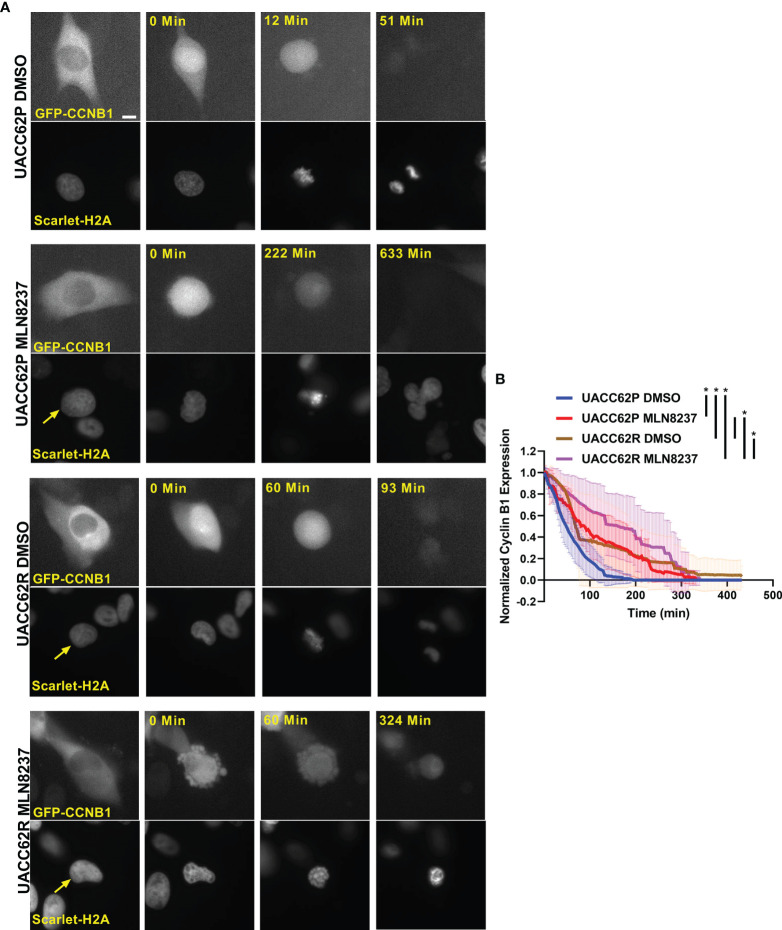
Differential CyclinB1 degradation rates in UACC62P/R cells treated with AURKi. **(A)** Representative images of EGFP-CyclinB1 and mScarlet-H2A in DMSO or MLN8237-treated UACC62P/R cells. Scale bar = 10 µM. **(B)** Quantification of single cell kinetics of EGFP-CyclinB1 expression levels in DMSO or MLN8237-treated UACC62P/R cells was performed as described in *Materials and Methods*. The thick lines represent the mean EGFP-CyclinB1 expression across all cells, and the thin lines represent the standard deviation. At least 8 cells were analyzed per treatment condition. The area under each curve was calculated, and significant differences in curve areas were computed with a one-way ANOVA with a Šídák’s multiple comparisons test. Statistical significance (p < 0.001) is indicated by an * near the corresponding figure label.

Degradation of Cyclin B1 drives the exit of cells from mitosis. In arrested cells, however, a failure to reduce Cyclin B1 levels below a critical threshold can result in cells undergoing mitotic slippage leading to greater than 2n DNA content and polyploid nuclei (21). We therefore hypothesized that the propensity of UACC62P cells, but not UACC62R cells, to undergo mitotic slippage upon treatment with inhibitors might be due to differences in the levels of Cyclin B1 at the mitotic spindle checkpoint. To explore this idea, we engineered UACC62P/R cells to stably express EGFP-CCNB1 (Cyclin B1) along with mScarlet-H2A to simultaneously monitor in real time both mitotic progression and Cyclin B1 levels in individual cells by live cell imaging. EGFP-Cyclin B1 expression and localization mirrors that of endogenous Cyclin B1 (27) and expression of EGFP-Cyclin B1 does not have a significant effect on cell cycle progression or expression of cell cycle-related genes (28). Levels of endogenous Cyclin B1 were slightly elevated in UACC62R cells compared with UACC62P cells, and in both cell lines expression of EGFP-Cyclin B1 was low relative to endogenous Cyclin B1 (**Figure S6**). There was also no difference in Cyclin A1 expression between the UACC62P and UACC62R cells (**Figure S6**). Prior to initiation of mitosis, EGFP-Cyclin B1 is sequestered in the cytosol in DMSO-treated UACC62P cells and then rapidly co-localizes with mScarlet-H2A upon chromosome condensation and nuclear envelope breakdown (**Figure 3A**). The DMSO-treated UACC62R cells displayed kinetics of EGFP-Cyclin B1 expression similar to that of DMSO-treated UACC62P cells (**Figure 3B**). In response to treatment with the AURKi, MLN8237, the levels of Cyclin B1 in UACC62R cells gradually reduced to approximately 50% of their original levels. In contrast, in the UACC62P cells treated with MLN8237, there was approximately a 90% reduction in the levels of EGFP-Cyclin B1, which could allow these cells to undergo mitotic slippage. Taken together, these data suggest that differential regulation of Cyclin B1 degradation dictates whether MLN8237-treated melanoma cells undergo mitotic slippage or prolonged cell cycle arrest and subsequent loss of viability.

### Increased Sensitivity of BRAFi-resistant M229R Cells to Chk1/2 Inhibitors

**Figure 4:**
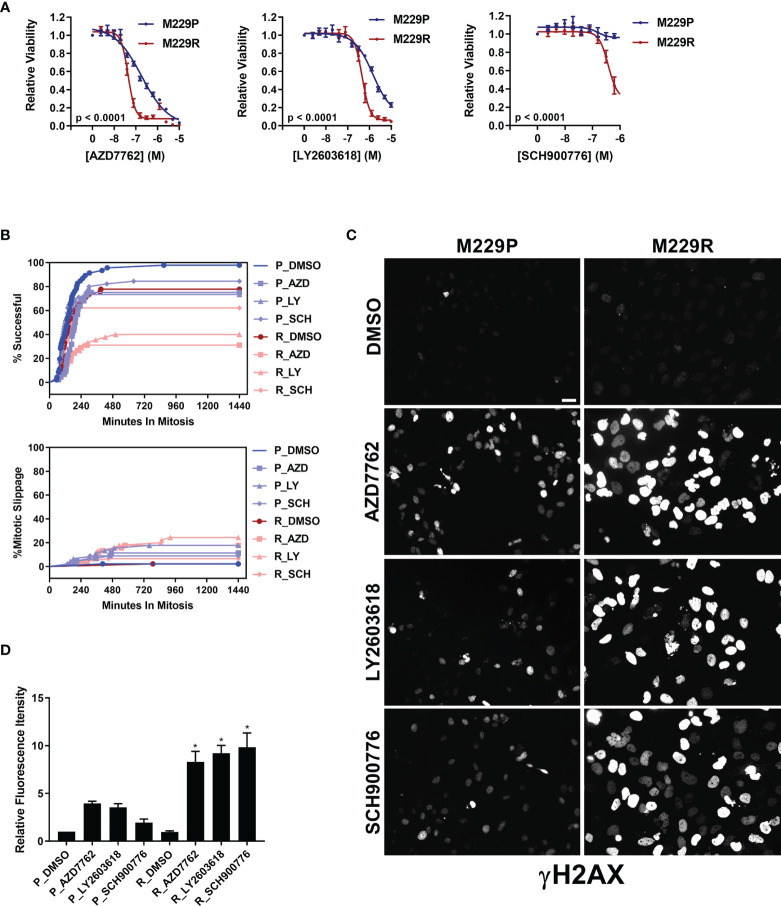
M229R cells are vulnerable to Chk1/2 inhibitors. **(A)** M229P/R cells were seeded into 384-well plates and treated with AZD7762, LY2603618, and SCH900776 as indicated. After 72 h, viability was measured as described in *Materials and Methods*. Data are represented as mean ± SE of the technical replicate averages for each of the biological replicates (n = 3). IC_50_ and E_max_ values are listed in **Supplementary Table S3**. **(B)** M229P/R cells were engineered to express mScarlet-H2A and EGFP-TUBA1B as described in the *Materials and Methods*. Cells were seeded into glass-bottom 96-well plates and the next day the cells were treated with 100 nM AZD7762, 1 µM LY2603618, or 1 µM SCH900776. Mitotic rate/outcome was measured on the Cytation 3 microscope setup as described in *Materials and Methods*. At least 40 cells were analyzed per treatment condition. **(C)** M229P/R cells were treated with 100 nM AZD7762, 1 µM LY2603618, or 1 µM SCH900776 for 24 h. The cells were subsequently fixed and stained with an antibody raised against p-γH2AX. Scale bar = 10 µM. **(D)** Quantification of γH2AX from the experiment in **Figure 4C** was as described in *Materials and Methods*. Statistical analysis was performed with one-way ANOVA analysis, * indicates p < 0.01 vs the M229R DMSO group. None of the compound-treated M229P groups was statistically significant in comparison to M229P DMSO. Data are represented as mean ± SE for n = 3 biological replicates. IC_50_ and E_max_ values are listed in (**Supplementary Table S3**).

Our compound screen revealed that vemurafenib resistance led to increased sensitivity to inhibitors of AURK, PLK, tubulin, and kinesin in UACC62 and M238 melanoma cells, whereas M229R cells showed increased sensitivity to 3 different Chk1/2 inhibitors compared with M229P cells (**Figure S2** and see **Table 1**). In a follow-up concentration response assay, we confirmed that the three Chk1/2 inhibitors selectively targeted M229R cells over the vemurafenib sensitive parental cell line (**Figure 4A**). Similar to our findings with the AURK/PLK/tubulin/kinesin inhibitors in the other melanoma cells, these inhibitors selectively reduce viability of BRAFi resistant cells compared with BRAFi sensitive parental cells, but do not re-sensitize to or have synergy with vemurafenib (**Figure S7**). While the mitotic success rate in response to Chk1/2 inhibitor treatment was reduced in M229R cells compared with M229P cells, the fraction of cells undergoing mitotic slippage was the same in M229P and M229R cells (**Figure 4B**). After 240 min approximately 70% of compound-treated M229P cells had completed mitosis whereas, depending on the Chk1/2 inhibitor used, only 30- 60% of M229R cells had successfully completed mitosis. These data suggest that while M229R cells are also differentially sensitive to mitotic disrupters, in this case Chk1/2 inhibitors; however, their increased vulnerability compared to M229P cells appears to be due to a mechanism distinct from mitotic slippage. Under physiological conditions Chk1/2 activation monitors DNA fidelity during replication and in response to DNA damage, ultimately preventing premature entry into mitosis (29). Chk1/2 inhibition would be expected to result in the accumulation of DNA damage, ultimately leading to failure in mitosis. Treatment with any of three structurally distinct Chk1/2 inhibitors resulted in increased γH2AX staining in M229R cells over M229P cells (**Figure 4C, D** and **Figure S8**). The increased DNA damage is probably not due to elevated ROS levels, since basal ROS was not elevated in M229R cells (**Figure S3**). Overall, these data suggest that Chk1/2 inhibitors selectively induce the accumulation of DNA damage in M229R cells, ultimately leading to a high rate of mitotic failure. This accumulation of DNA damage is likely not a direct effect of Chk1/2 inhibitors, but rather inhibition of Chk1/2 in these cells prevents the repair of DNA damage which is introduced from another source. This, in turn, suggests that the M229R cells may be more prone to DNA damage which would ultimately lead to cell cycle arrest, as has been previously demonstrated with Chk1/2 inhibitors (30).

## Discussion

In this study we discovered, through screening of a mechanistically defined library, that BRAFi resistance in melanoma cell lines is accompanied by increased sensitivity to a broad class of mitotic disrupters, including AURK, PLK, tubulin, and kinesin inhibitors or, in the one resistant line, to Chk1/2 inhibitors. Two lines (UACC62 and M238) showed sensitivity to the former agents and one (M229) to the latter, while a fourth line (M249) which has a different resistance mechanism (acquired NRAS mutation) had no obvious vulnerabilities.

The sensitivity to mitotic inhibitors is associated with an inability of the BRAFi-resistant cells to undergo mitotic slippage. Mitotic slippage is a well characterized resistance mechanism for multiple classes of mitotic inhibitors, including those which disrupt tubulin polymerization/depolymerization (31–33). Our data also support the idea that the vulnerability of UACC62R cells to mitotic arrest is due to the absence of mitotic slippage upon treatment with the mitotic inhibitor MLN8237. This results from differential Cyclin B1 degradation, since Cyclin B1 degradation is a key initiating event during mitotic slippage. In mitosis, the anaphase-promoting complex (APC) is activated during metaphase and targets Cyclin B1 for degradation (34). Upon treatment with the AURKi MLN8237, both parental and BRAFi resistant UACC62 cells appeared to arrest in prophase/prometaphase with condensed chromosomes but without alignment of the chromosomes along the metaphase plate (**Figure 2C**). These data would suggest that the APC is still inactivated in these cells, which should prevent the degradation of Cyclin B1 (35). It is possible that a low level of APC activation is present in UACC62P, but not UACC62R, cells, which would result in the gradual degradation of Cyclin B1 and eventual mitotic slippage. Another possibility is that the APC may be fully inactivated in both UACC62P and UACC62R cells, but APC-independent Cyclin B1 degradation mechanisms could have higher activity levels UACC62P cells. Further clarification of these mechanisms will be important since they could serve as biomarkers for identifying tumors which are more responsive to disruption of mitosis.

One interesting observation in our dose response validation studies of the mitotic inhibitors was that in many cases there was no difference in IC_50_ between the parental and resistant cells. Rather, E_max_ between the parental and resistant cells was altered. The likely explanation for the reduced E_max_ is that a significant fraction of the parental cells are able to undergo mitotic slippage and survive compound treatment. Consistent with this hypothesis, the fraction of UACC62P cells that undergo mitotic slippage in response to a mitotic inhibitor corresponds well with the E_max_ of the dose response curve for that mitotic inhibitor.

Another BRAFi-resistant cellular model, M229R, acquired increased sensitivity to Chk1/2 inhibitors. While the molecular mechanism governing this selectivity is different from that of UACC62P/R cells, the commonality is that both cellular models are vulnerable to inhibitors which directly or indirectly disrupt mitosis. Chk1/2 inhibitors induced more severe accumulation of the DNA damage marker γH2AX in M229R cells than in M229P cells. This could indicate that excessive DNA damage is causing the M229R cells to arrest and ultimately die during mitosis. One possible explanation for the differential response to Chk1/2 inhibitors is functional redundancy between Chk1/2 and other DNA repair pathways in the parental M229 cells. For instance, if M229R cells are defective in other DNA repair mechanisms (such as PARP), this would increase their dependence on Chk1/2 for DNA repair, ultimately resulting in an elevated accumulation of DNA damage in Chk1/2 inhibitor-treated M229R cells. Consistent with this idea, PARP inhibitors have been shown to synergize with CHK1 inhibitors (36). This mechanism would also explain why there is no difference in γH2AX staining in DMSO-treated M229R cells, since in the absence of Chk1/2 inhibitors M229R cells would still retain the ability to perform DNA repair. An analogous mechanism explains the elevated sensitivity of BRCA1/2-mutant tumors to PARP inhibitors in the classic example of synthetic lethality (37).

Previous studies found that enhanced replication stress increases the response of melanoma cells to CHK1 inhibitors *in vivo* (38). This might suggest that enhanced replication stress in M229R cells could explain the differential response to CHK1 inhibitors. Enhanced oncogenic signaling, as can occur through abberant Myc or Ras activation, increases replication stress in cancer cells (39). So, it is possible that if these signaling pathways, or other signaling pathways which also drive proliferation, are differentially activated in M229R cells they may dictate the unique response to CHK1 inhibitors. Consistent with the idea that replication stress dictates CHKi response, silencing of multiple DNA polymerase isoforms, including POLA1, POLE, and POLE2, enhances replication stress and increases CHK1i sensitivity in lung and colorectal cancer cell lines (40).

In summary, using a drug repurposing screening approach, we have identified acquired pharmacological vulnerabilities to compounds that result in mitotic disruption in three different poorly differentiated BRAFi resistant human melanoma cell lines which also exhibit enhanced RhoA/C signaling (23). In contrast, no enrichment of any compound class showed selective toxicity in a melanoma line that developed BRAFi resistance through acquisition an NRAS mutation and lacks a de-differentiated phenotype. This observation suggests that melanoma cells and tumors whose resistance is associated with a de-differentiation phenotype are more vulnerable to compounds which disrupt mitosis. If biomarkers for response to anti-mitotic agents can be established, it may be possible to identify a subset of resistant tumors which are vulnerable to second-line therapy with these classes of approved drugs.

While we did not observe synergy between BRAF inhibitors and mitotic inhibitors in BRAFi-resistant cells, the combination of these agents still warrants further investigation, since other studies have reported an additive effect of the combination of CHK1/2, Aurora kinase, or PLK inhibitors and BRAF inhibitors *in vitro* and *in vivo* (41). An expanded analysis that profiles the response of BRAFi-resistant melanoma cells to mitotic inhibitors is necessary to fully elucidate the molecular features predictive of response to the combination of BRAF inhibitors and mitotic inhibitors. The finding that BRAFi-resistant cells are more sensitive to mitotic inhibitors suggests that tumors may be more sensitive to these agents after developing resistance to MAPK pathway inhibitor therapy. However, another intriguing possibility is that BRAF and mitotic inhibitors could be combined at the onset of treatment to prevent or forestall the development of drug resistance. This is especially true if mechanisms of resistance to BRAF/MEK inhibitors are mutually exclusive to mechanisms of resistance for mitosis inhibitors.

## Data Availability Statement

The raw data supporting the conclusions of this article will be made available by the authors, without undue reservation.

## Author Contributions

SM, BF, TD, and MA conducted experiments. JS provided experimental resources and expertise. SM, TD, SC, RN, KG designed the study. SM, JS, RN, and KG acquired funding for this study. All authors contributed to the article and approved the submitted version.

## Funding

MSU Gran Fondo Skin Cancer Research Fund (RRN), MSUFCU Aitch Foundation Fellowship (SM), NIH F31 CA232555 (SM), NIH R25 HL103156 (JS), NIH R00 GM120386 (JS), Damon Runyon Cancer Research Foundation DFS-24-17 (JS). MA was supported by NIH/NHLBI-MSU BRUSH program (5 R25 HL103156).

## Conflict of Interest

The authors declare that the research was conducted in the absence of any commercial or financial relationships that could be construed as a potential conflict of interest.

## Publisher’s Note

All claims expressed in this article are solely those of the authors and do not necessarily represent those of their affiliated organizations, or those of the publisher, the editors and the reviewers. Any product that may be evaluated in this article, or claim that may be made by its manufacturer, is not guaranteed or endorsed by the publisher.
