# Supplementary material for: BRAF Inhibitor Resistance Confers Increased Sensitivity to Mitotic Inhibitors
Source: Front Oncol. 2022 Apr 4;12:766794. doi: 10.3389/fonc.2022.766794 (PMC9015667; doi:10.3389/fonc.2022.766794)
Supplement: Supplementary file 1 [file DataSheet_1.docx]

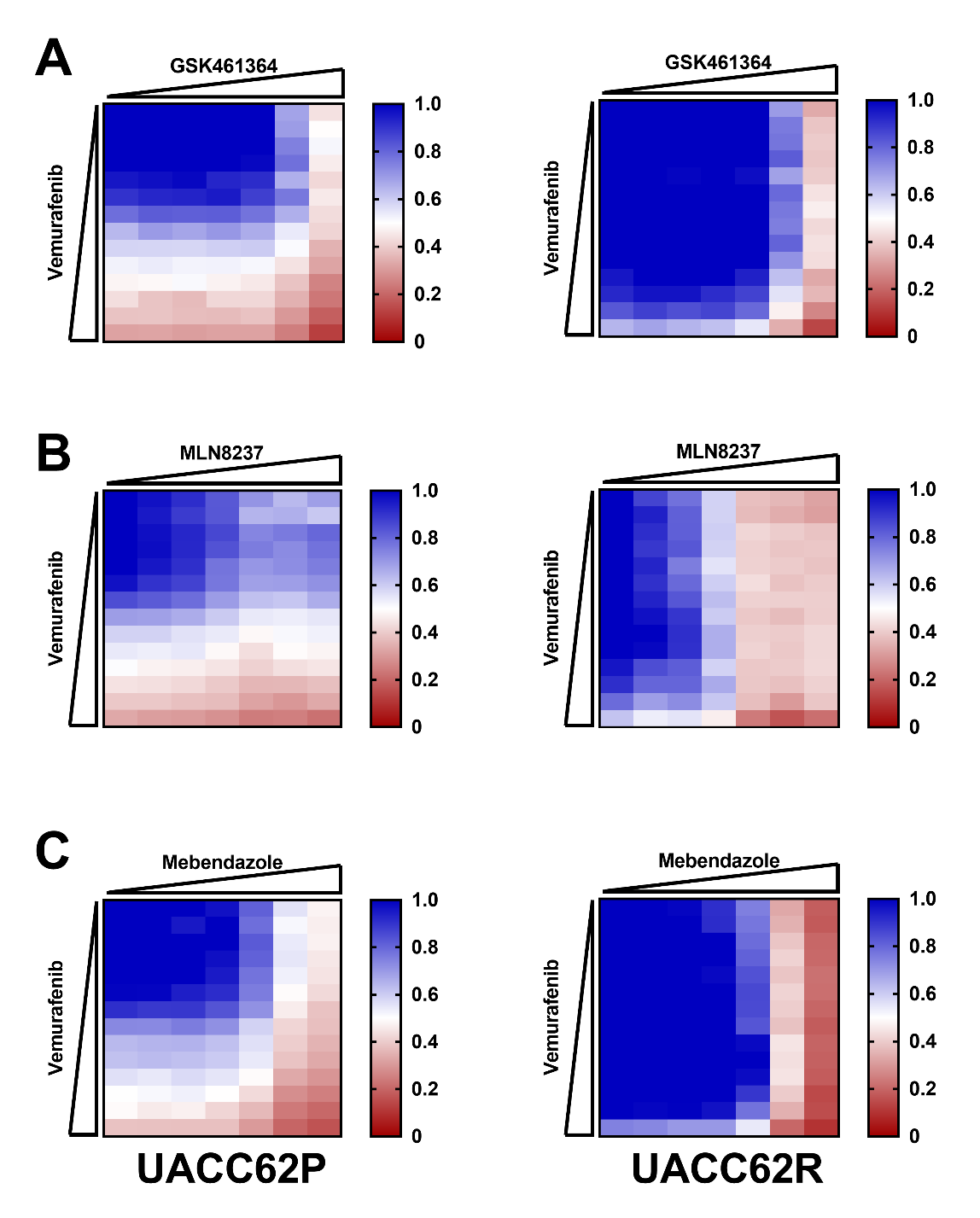


**Figure S1. AURK, PLK, and Tubulin inhibitors do not synergize with vemurafenib in UACC62P/R cells.** UACC62P/R cells were seeded into 384-well plates at a density of 1,000 cells/well. The next day the cells were treated in a concentration response matrix with a top concentration of 10 µM for all compounds and a ½ dilution series. Viability was analyzed as described in the Materials and Methods section. Data is expressed as relative viability wherein a value of 1 (blue) indicates 100% viability and a value of 0 (red) indicates 0% viability. **A.** UACC62P/R cells were treated with a GSK461364 x Vemurafenib concentration response matrix. **B.** UACC62P/R cells were treated with a MLN8237 x Vemurafenib concentration response matrix. **C.** UACC62P/R cells were treated with a Mebendazole x Vemurafenib concentration response matrix. This experiment was repeated with n = 3 biological replicates. The data in these matrices represent the average compound response across all experimental replicates.


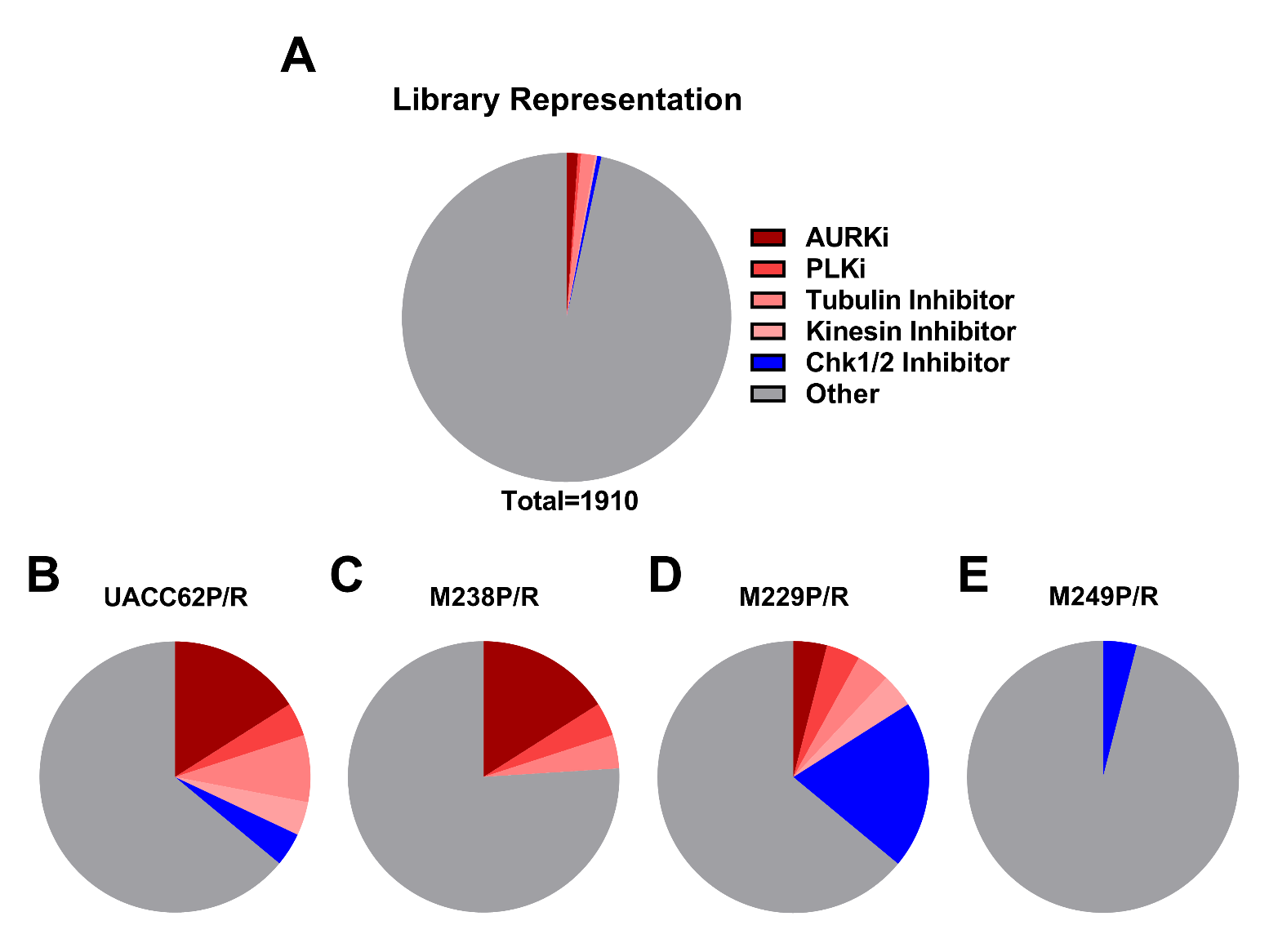
**Figure S2. Identification of compound classes which are selective for BRAFi-resistant cells. A)** Overall compound representation in the MIPE library. AURKi, PLKi, Tubulin inhibitors, Kinesin inhibitors, and Chk1/2 inhibitors are highlighted. Compound class enrichment for the top 25 most selective compounds in **B)** UACC62P/R cells, **C)** M238P/R cells, **D)** M229P/R cells, and **E)** M249P/R cells.


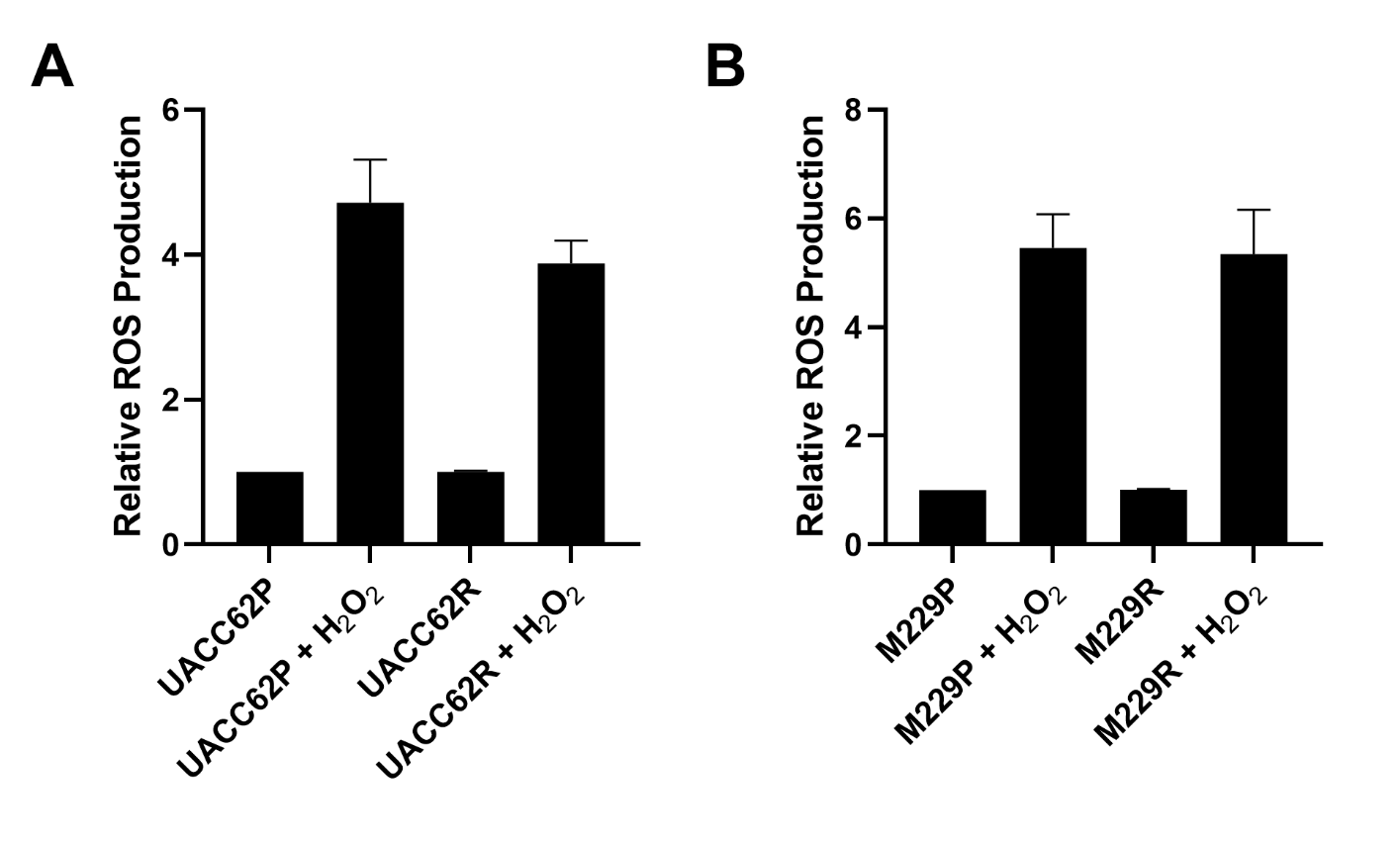


**Figure S3. ROS production is not altered in BRAFi-resistant cells.** **A)** UACC62P/R and **B)** M229P/R cells were seeded into 96-well plates. The next day the cells were treated with H_2_O_2_ and the ROS assay was performed as described in the Materials and Methods with cells treated with H_2_O_2_ serving as a positive control. Relative ROS production is normalized to ROS levels in UACC62P (panel A) or M229P (panel B). This experiment was repeated with n = 3 technical replicates and n = 3 biological replicates.


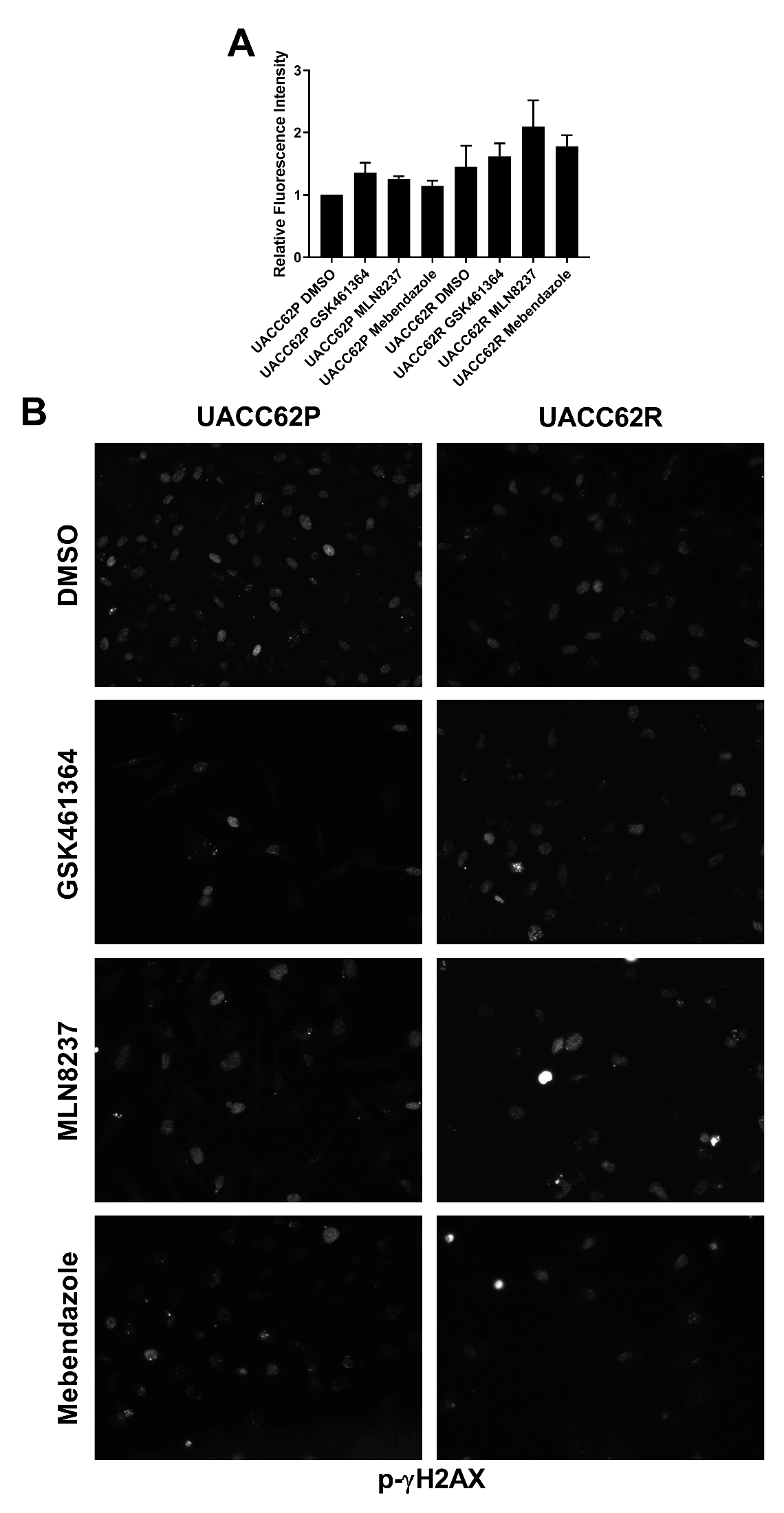


**Figure S4. p-γH2AX staining is not altered in compound-treated UACC62P/R cells. A**) UACC62P/R cells were treated with 1 µM GSK461364, MLN8237, or Mebendazole for 24 h. The cells were fixed and stained with a p-γH2AX antibody and quantified as described *Materials and Methods*. **B)** Representative immunofluorescence images. This experiment was repeated with n = 3 biological replicates.


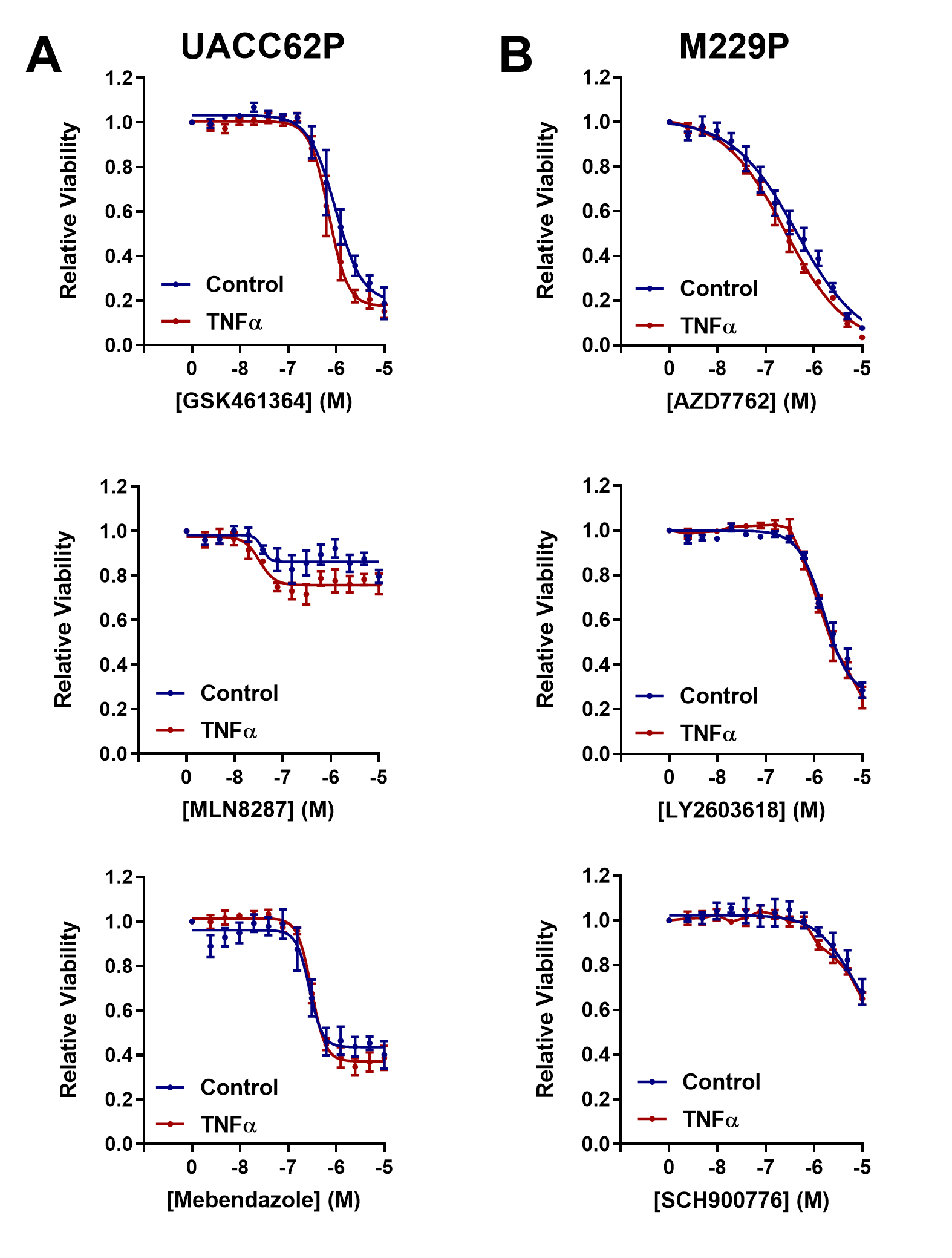


**Figure S5. TNFα does not alter AURK, PLK, Tubulin, or Chk1/2 inhibitor sensitivity. A)** UACC62P or **B)** M229P cells were seeded into 384-well plates at a density of 1,000 cells/well. The next day the cells were treated -/+ 10 ng/mL TNFα and a concentration gradient of the indicated compound. Viability was measured and quantified as described in the Materials and Methods section. This experiment was repeated with n = 3 technical replicates and n = 3 biological replicates.


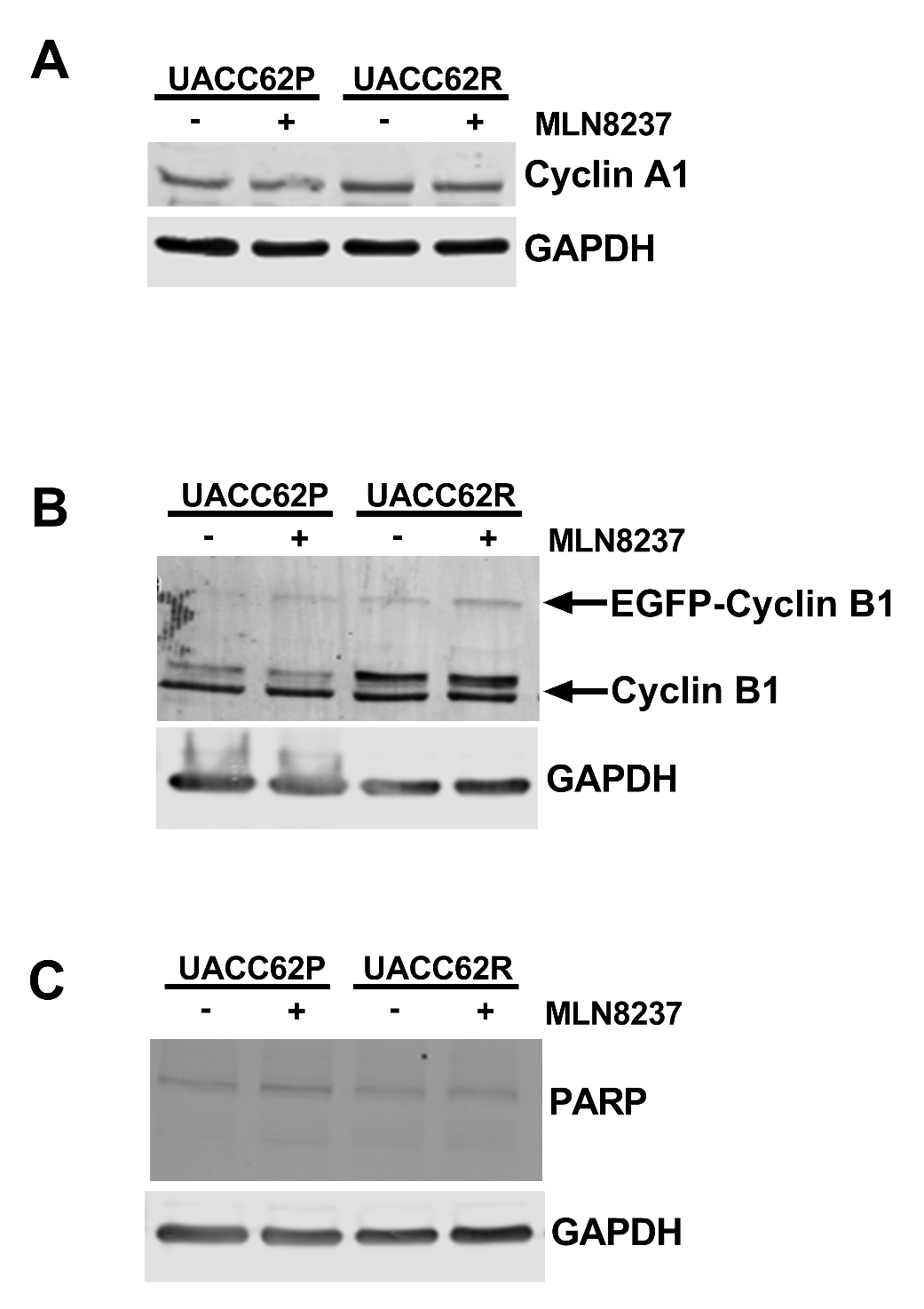


**Figure S6.** **Cyclin B1, Cyclin A1, and PARP levels in MLN8237-treated UACC62P/R cells.** UACC62P and UACC62R cells were engineered to express EGFP-Cyclin B1 as described in the Materials and Methods section. The cells were treated with 1 µM MLN8237 for 24 h before cellular lysates were collected. Immunoblots were performed to measure levels of **(A)** Cyclin A1, **(B)** Cyclin B1, and **(C)** PARP. GAPDH was using as a loading control.


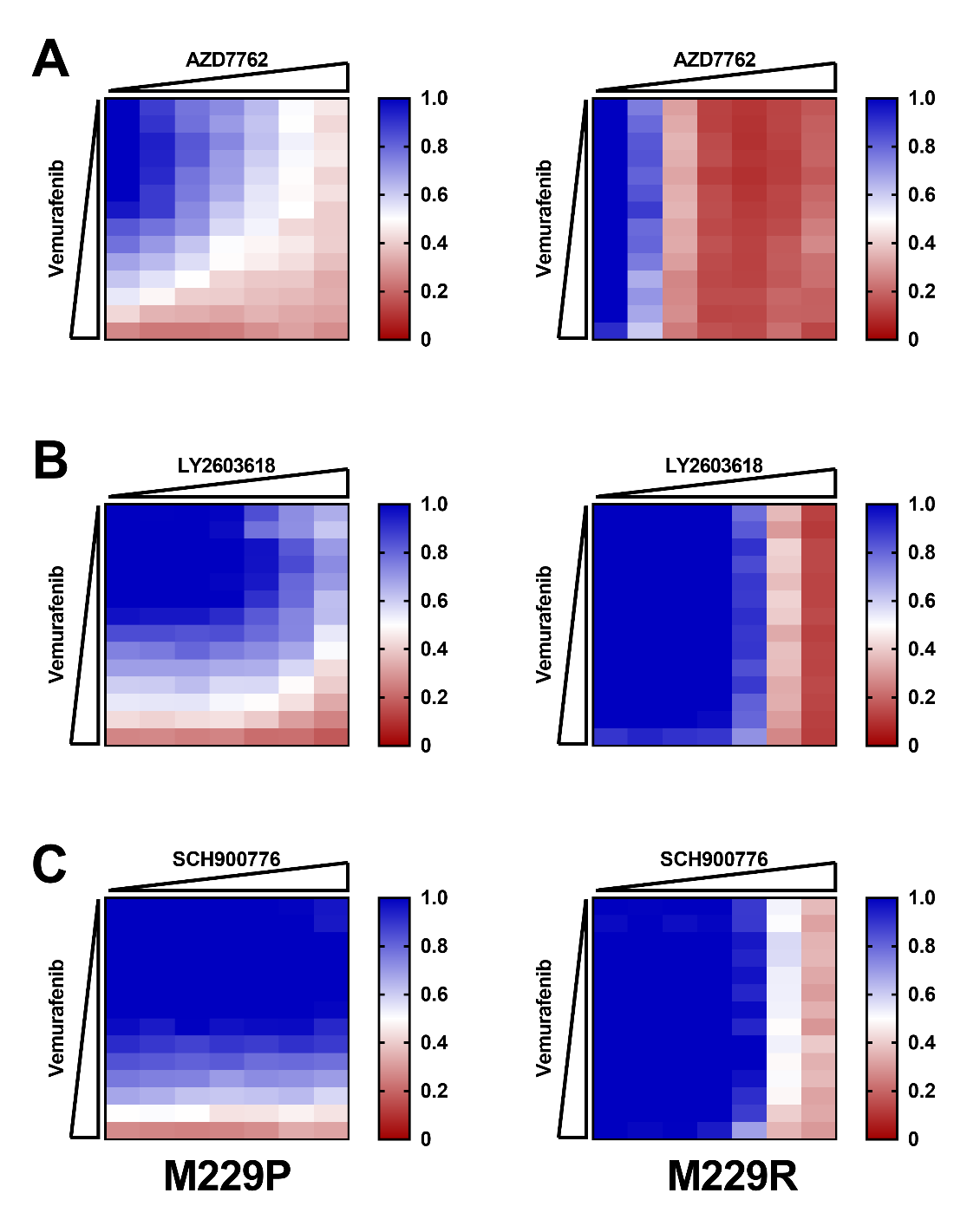


**Figure S7. Chk1/2 inhibitors do not synergize with vemurafenib in M229P/R cells.** M229P/R cells were seeded into 384-well plates at a density of 1,000 cells/well. The next day the cells were treated in a concentration response matrix with a top concentration of 10 µM for all compounds and a ½ dilution series. Viability was analyzed as described in the Materials and Methods section. Data is expressed as relative viability wherein a value of 1 (blue) indicates 100% viability and a value of 0 (red) indicates 0% viability. **A.** M229P/R cells were treated with a AZD7762 x Vemurafenib concentration response matrix. **B.** M229P/R cells were treated with a LY2603618 x Vemurafenib concentration response matrix. **C.** M229P/R cells were treated with a SCH900776 x Vemurafenib concentration response matrix. This experiment was repeated with n = 3 biological replicates. The data in these matrices represent the average compound response across all experimental replicates.


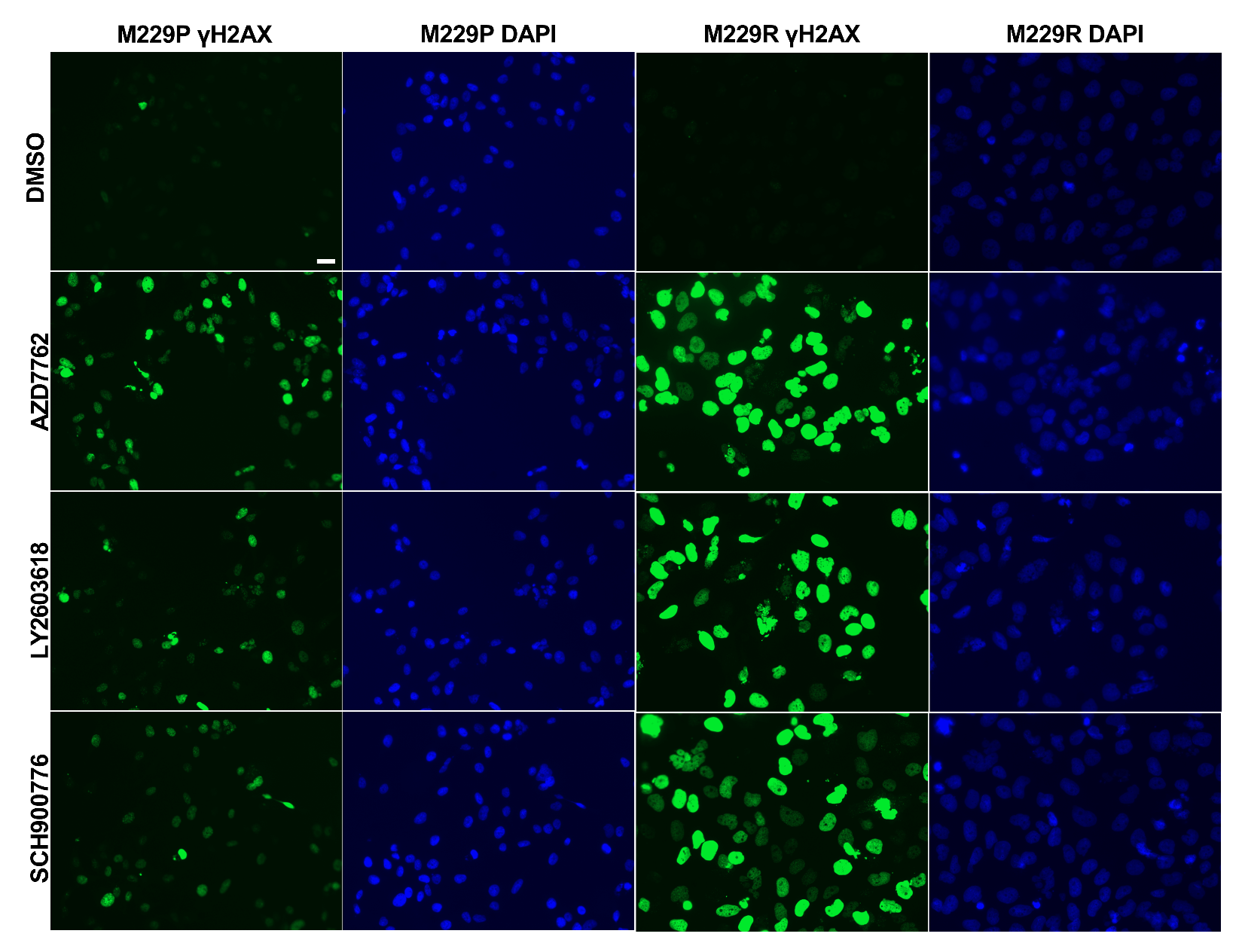


**Figure S8: Chk1/2 inhibitors increase γH2AX staining in M229P/R cells.** M229P/R cells were treated with 100 nM AZD7762, 1 µM LY2603618, or 1 µM SCH900776 for 24 h. The cells were subsequently fixed and stained with an antibody raised against p-γH2AX. Scale bar = 10 µM. These images are color versions of the images in Fig. 4C. Scale bar = 10 µM.
